# Revision on the Genus *Paris* in Thailand, with a New Species *Paris siamensis*

**DOI:** 10.3390/plants12030430

**Published:** 2023-01-17

**Authors:** Saroj Ruchisansakun, Supajit Sraphet, Chatchai Yothawut, Chompunooch Thamanukornsri, Nawarat Suksee, Panida Kongsawadworakul, Nattaya Srisawad, Nicha Thawara, Puangpaka Umpunjun, Supaporn Rodpradit, Winai Sangkaew, Kanokporn Triwitayakorn

**Affiliations:** 1 Department of Plant Science, Faculty of Science, Mahidol University, Ratchathewi, Bangkok 10400, Thailand; 2 Institute of Molecular Biosciences, Mahidol University, Salaya, Nakhon Pathom 73170, Thailand; 3 Doi Phu Kha National Park, Pua Nan 55120, Thailand; 4 Institute of Science, School of Mathematics, Suranaree University of Technology, Mueang Nakhon Ratchasima, Nakhon Ratchasima 30000, Thailand; 5 Queen Sirikit Botanic Garden, Botanical Garden Organization, Mae Rim, Chiang Mai 50180, Thailand; 6 Faculty of Agricultural Production, Maejo University, San Sai District, Chiang Mai 50290, Thailand

**Keywords:** *Paris chinensis*, Melanthiaceae, threatened species, cryptic taxa, *Paris polyphylla*

## Abstract

The genus *Paris* is an important and confusing taxon due to high variation within species, and differences between species are sometimes difficult to delimit. Thus, the status of some taxa has changed over time. To clarify the status of *Paris* species for plant conservation and effective management of this genus in Thailand, we performed an intensive survey in northern Thailand, studied morphological characteristics, and constructed a molecular phylogenic tree, which we compared to recently published results of this genus. Our results indicate that there are two species in Thailand: *P. yunnanensis* and a new species, *P. siamensis*. Detailed descriptions, illustrations, and the phylogenetic position of these two species are provided here.

## 1. Introduction

Plants in the genus *Paris* L. [1] are important Chinese medicinal plants [2]. In recent years, these plants have been collected in Thailand and exported to China, causing them to become extremely threatened [2]. For the conservation and effective management of this genus, intensive taxonomic studies are required. The morphological characteristics of this genus have been reported to be distinct from other genera [2]. However, broad variation within species (e.g., in the shape and size of each plant part) has been observed, resulting in difficulties when identifying plants at the species level.

In the flora of Thailand [3], only one species of the genus *Paris* is reported in Thailand: *P. chinensis* Franch. [4] (=*Daiswa polyphylla* var. *chinensis* (Franch.) M. N. Tamura. in the book). However, in the taxonomic revision of *Paris*, Ji [2] mentioned that two species were found in Thailand: *P. chinensis* and *P. caobangensis* [5]. From our revision using an intensive morphological and molecular examination of fresh specimens in Thailand, we found that *P. yunnanensis* was distributed in many provinces in Northern Thailand. However, populations in Nan province seemed to differ from other populations but were unable to be confidently identified as any of the species reported in the recent revision [2]. Hence, both morphological and molecular methods were applied to identify and confirm the status of *Paris* in northern Thailand [6,7,8].

## 2. Results

### 2.1. Taxonomic Treatment

#### 2.1.1. *Paris yunnanensis* Franch. Mem. Philom. Cent. (Paris) 24: 290. 1888. (Figure 1)

*Paris yunnanensis* Franch; C. H. Wright, Journ. Linn. Bot. 36:145. 1903.—*Paris polyphylla* Smith var. *yunnanensis* (Franch.) Hand.-Mazz., Symb. Sin. 7: 1216. 1936; Hara, Journ. Fac. Sci. Univ. Tokyo. Sect. 3, 10 (10): 154. 1069; Wang et Tang, Fl. Reip. Pop. Sin. 15: 95. 1978; H. Li, Bull. Bot. Res. Harbin 6 (1): 119. 1986; H. Li, The Genus *Paris* (Trilliaceae) 35. 1998; S. Y. Liang et V. G. Soukup, Fl. Chin. 24: 90. 2000.—*Daiswa yunnanensis* (Franch.) Takht., Brittonia 35 (3): 257. 1983; B. Mitchell, Plantsman 10 (3): 185. 1988. Type: China, Yunnan, Eryuan, 2000 m, J. M. Delavay 2227 (holotype P!).

*Paris christii* Lévl., Bull. Acad. Inter. Geogr. Bot. 12: 255. 1903. Type: China, Guizhou, Bodinier s. n. (holotype E!). (synonymized in [9]).

*Paris mercieri* Lévl., Bull. Acad. Int. Géogr. Bot. 12: 255. 1903. Type: China, Guizhou, Guiyang, 17 July 1898, Bodinier 1635 (holotype E!). (synonymized in [9]).

*Paris franchetiana* Lévl., Bull. Acad. Int. Géogr. Bot. 12: 255. 1903. Type: China, Guizhou, Bodinier 712 (holotype E!). (synonymized in [9]).

*Paris cavaleriei* Lévl. et Vaniot, Nouv. Contrib. Liliac. Chine 24: 354. 1906. Type: China, Guizhou, Longli, 13 June 1902, Cavalerie 1310 (holotype E!). (synonymized in [9]).

*Paris gigas* Lévl. et Vaniot, Nouv. Contrib. Liliac. Chine 24: 354. 1906. Type: China, Guizhou, Guiding, 23 November 1902, Cavalerie 729 (holotype E!). (synonymized in [9]).

*Paris pinfaensis* Lévl., Repert. Spec. Nov. Regni Veg. 6: 265. 1909. Type: China, Guizhou, Guiding, June 1907, Cavalerie 2023 (holotype E!, isotype GH, K!). (synonymized in [9]).

*Paris aprica* Lévl., Repert. Spec. Nov. Regni Veg. 6: 265. 1909. Type: China, Guizhou, Guiding, 25 June 1907, Cavalerie 3023 (holotype E!, isotype E!). (synonymized in [9]).

*Paris atrata* Lévl., Repert. Spec. Nov. Regni Veg. 12: 536. 1913. Type: China, Yunnan, Ninglang, Maire s. n. (holotype E!). (synonymized in [9]).

*Paris polyphylla* Smith var. *nana* H. Li, Bull. Bot. Res. Harbin 6 (1): 123. 1986. Maity et al., J. Bot. Soc. Bengal 68: 117. 2014. Type: China, Sichuan, Yibin, 07 July 1977, Yibin Drug Inspection Institute Yi428 (KUN). (synonymized in [2]).

*Paris daliensis* H. Li et V. G. Soukup, Act. Bot. Yunnanica, Supp. v: 15–16, Figure 3. 1992; H. Li, The Genus Paris (Trilliaceae) 32. 1998; S. Y. Liang et V. G. Soukup, Fl. Chin. 24: 89–90. 2000. Type: China, Yunnan, Dali, 2600 m, 06 Nov. 1986, H. Li & V. G. Soukup 1098 (holotype KUN, isotype CINC). (synonymized in [2]).

*Paris birmanica* (Takht.) H. Li et H. Noltie., Edinb. J. Bot. 54 (3): 351–352. 1997; H. Li, The Genus Paris (Trilliaceae). 28. 1998. *Daiswa birmanica* Takht., Brittonia 35 (3): 259, Figure 2. 1983; B. Mitchell, Plantsman 10 (3): 169. 1988; Type: Myanmar, Maymyo, 22 June 1913, Lace John Henry 6233 (holotype E!, isotype E!). (synonymized in [2]).

*Paris polyphylla* Smith var. *emeiensis* X. H. Yin, H. Zhang & D. Xue, Acta Phytotaxon. Sin. 45 (6): 822–827. 2007. Type: China, Sichuan, Mt. Emei, 1900 m, 27 April 2006, Yin XH et al. 06121 (holotype SZ, isotype, CDBI). (synonymized in [2]).

Perennial herb, (20–)27.7–121.5(–130) cm tall. Rhizome thick, cylindrical, conical, to C-shaped, 4–19 cm long, 1.5–7(–10) cm in diam., glabrous, with 2–12 scars on the flowering state. *Aerial stems* 15–105 cm long, 0.5–1.8 cm in diam., purple to green, or green at the upper part, glabrous to remotely short spiny. *Leaves* 5–11; *petiole* 1–6.5(–8) cm long, 1.5–4 mm in diam., purple above, glabrous; *lamina* 7.7–25 × 4.3–10.5(–15) cm, obovate, obovate, to oblanceolate, base cuneate, apex acute, width/length ratio 0.29–0.71: lateral veins two pairs. *Flower* solitary, terminal; *pedicel* (2.5–)5.3–24(–30) cm long, 1.5–6 mm in diam., green to purple with green at the upper part, glabrous; *Sepals* (3–)4–8(–10), leaf-like, 4.2–14.5 × 1.3–5 cm, lanceolate to elliptic, width/length ratio 0.17–0.44, base cuneate to obtuse, apex acute, green, glabrous; *petals* (3–)5–8(–10), (1.2–)3–7.5(–8.5) cm × 0.5–2(–2.5) mm, linear, protruding above the sepal, yellow to green: upper part (0.8–)1.5–2(–2.5) cm × (0.5–)1–2(–3) mm, yellow, petals/sepal length ratio (0.19–)0.33–0.89; *Stamen* (8–)10–22(–25), stamens/petals number ratio (1.83–)2–3.2(–4.4): filaments (1–)2–6(–7) mm long, (0.3–)1(–2) mm in diam.: anthers 7–13(–17) mm long; free portion of connective up to (0.2–)0.5–1.5 × (0.2–)1–1.5 mm, green to yellow to brown, glabrous, anther/filament length ratio (1.43–)2–6. *Pistil* 1: style with an enlarged base: Stylar base purplish red to orange: Ovary 4–8(–10) angled, single-locular with parietal placentation: stigma (3–)4–7(–15), (2–)3–8(–12) mm long, strongly curved, orange to red. Fruits dehiscent capsule, angular, 4–8(–10) angled; immature fruit up to 27 mm long, up to 40 mm in diam., green to dark green, yellow at ovary base, glabrous. Seed white covered by red to orange: sarcotesta red to orange.

*Phenology*. Flowering April–August. Fruiting July–February.

*Distribution*. Myanmar (Mandalay and Shan). China (Chongqing, Guangxi, Guizhou, Sichuan, Tibet, Yunnan). Thailand (Northern).

*Additional Specimens Examined*. THAILAND. NORTHERN. Chiang Mai.

[Chiang Dao Distr., Doi Chaing Dao Wildlife Sanctuary, 1400 m alt., 6 February 2012, *V. Chamchumroon* et al. *V.C. 1764* (BKF141913); ibid., 1200 m alt., 6 December 1959, *T. Smitinand & E.C. abbe 6213* (BKF24723); ibid., 1020 m alt., 23 June 1998, *Khantchai 192* (BKF24021); ibid., 7 May 1999, *PS. Plernchit 1205* (BKF15756); ibid., 1400–1500 m alt., 16 December 1983, *N. Fukuoka & M. Ito T-35234* (BKF109875, L3915025); ibid., 22 December 1931, *K. Larsen* et al. *2919* (L.1460262); ibid., 22/12/1931, *Put s. n.* (BK25671). Chom Thong Distr., Bann Angka Noi, Doi Inthanon National Park, 1160 m alt., 4 August 2003, *TH. Wongprasert & S. Khaoiam 038–63* (BKF157006); ibid., 900 m alt., 26 June 1978, *C. Phengkai* et al., *4118* (BKF); Doi Inthanon National Park, Mae Pan waterfall, 1100 m alt., 16 October 1979, *T. Shimizu* et al. *18975* (BKF76677); Doi Inthanon, 1020 m alt., 22 July 1988, *M.N. Tamura T-60181* (BKF94753); ibid., 1150 m alt, 22 October 1999, *Th. Wongprasert s. n.* (BKF128662). Pha Mon, 2 January 1981, *Somkid 115* (BKF110099), Doi Inthanon National Park, Mae Klang Luang Village, 1615 m alt, 23 May 2011, *Pavlos Georgiadis 615* (L4345332), Mae Soi Ridge, Mae Soi Subdistr., Ban Bah Gluay (Meo village), 16 June 1991, *JF. Maxwell 91-551* (L3896551); ibid., 8 May 1992, *J.F. Maxwell 92-189* (L3893265); Doi Saket Distr., Thep Sadet, 1206 m alt, 16 July 2020, *S. Ruchisansakun* et al. *PJDSK63008* (QBG); *S. Ruchisansakun* et al. *PJDSK63010* (QBG); *S. Ruchisansakun* et al. *PJDSK63013* (QBG); *S. Ruchisansakun* et al. *PJDSK63014* (QBG); *S. Ruchisansakun* et al. *PJDSK63015* (QBG); *S. Ruchisansakun* et al. *PJDSK63018* (QBG); *S. Ruchisansakun* et al. *PJDSK63020* (QBG); *S. Ruchisansakun* et al. *PJDSK63021* (QBG); *S. Ruchisansakun* et al. *PJDSK63025* (QBG); *S. Ruchisansakun* et al. *PJDSK63026* (QBG); *S. Ruchisansakun* et al. *PJDSK63027* (QBG); *S. Ruchisansakun* et al. *PJDSK63028* (QBG); *S. Ruchisansakun* et al. *PJDSK63030* (QBG); *S. Ruchisansakun* et al. *PJDSK63032* (QBG); *S. Ruchisansakun* et al. *PJDSK63035* (QBG); *S. Ruchisansakun* et al. *PJDSK63037* (QBG); *S. Ruchisansakun* et al. *PJDSK63040* (QBG); *S. Ruchisansakun* et al. *PJDSK63044* (QBG); Chai Prakan Distr., Wiang Pha, 800 m alt., 11 November 2021, *S. Ruchisansakun* et al. *PW641111* (TR64) (QBG); Fang Distr., Doi Fa Hom Pok NP, 19 December 2010, *P. Phonsena* et al. *6673* (L3809213); Hod Distr., Mai Muang Nao Arboretum, Ban Mae Sanam Mai, Baw Salee Subdistr., 15 May 2001, *W. Sankamethawee 175* (L3809523); Mae Chaem Distr., Mae Suek, Ban Pha La Pi, 1541 m alt., 18 June 2022, *S. Ruchisansakun* et al. *PW650618* (QBG); *S. Ruchisansakun* et al. *PW650619* (QBG); Mae Taeng Distr., Pa Pae, 14 June 2020, *S. Ruchisansakun* et al. *PJMT63008* (QBG); *PJMT63010* (QBG); *PJMT63012* (QBG); *PJMT63013* (QBG); *PJMT63017* (QBG); *PJMT63018* (QBG); Mae Rim Distr., Pong Yaeng, Queen Sirikit Botanical Garden, 15 July 2020, *S. Ruchisansakun* et al. *PJHL63003* (QBG); *PJHL63004* (QBG); *PJHL63005* (QBG); *PJHL63006* (QBG); *PJHL63007* (QBG), *PJHL63008* (QBG); *PJHL63020* (QBG); *PJHL63021* (QBG); *PJHL63022* (QBG); Queen Sirikit Botanical Garden, 28 September 2005, *Tillich 5045* (BKF163774); Mae Rim Distr., Mae Sa valley, 1000 m alt. 17 May 1974, *J.K. Jackson 6052* (BKF58733); Mae Rim Distr., Saluang, Phraphutthabat Si Roi, 5 August 2020, *S. Ruchisansakun* et al. *PJPPB63012* (QBG); *PJPPB63013* (QBG); *PJPPB63014* (QBG); Mae Wang Distr., Mae Win, Ban Pah Kluai, 1110 m alt, 20 June 2022, *S. Ruchisansakun* et al. *PW650620* (QBG); Mueang Chiang Mai Distr., Mueang Chiang Mai Distr., Doi Pui, ca. 1500 m alt., 14 October 1979, *T. Shimizu* et al. *18637* (BKF76676); Doi Suthep, 25 July 1991, *Soradet SS*. *159* (BKF4372); ibid., 24 April 1977, *Sin Boonchu 1860* (BKF112159); ibid., 5 December 1987, *J.F. Maxwell 87-1542* (L1460259, L1460260); ibid., 3 May 1958, *T. Sorensen 3229* (L1460261); Suthep-Pui National Park, 1300–1500 m alt., 22 October 1988, *C. Phengklai* et al. *6541* (BKF91009); Payap, Doi Buak Ha, 1150 m alt., 29 November 1965, *E. Hennipman 3165* (BKF39737, L1449986, L1449987); Samoeng Distr., Maerim-Samueng road, Maeo village, 900 m alt., 28 December 1987, *R. Pooma 53* (BKF99839)]. Chiang Rai [Mae Fa Luang Distr., Mae Salong Nok, 1207 m alt., 22 April 2022, *P. Umpunjun* et al. *PW650422* (QBG), Mueang Chiang Rai Distr., Huai Chomphu, *Saroj Ruchisansakun,* et al. *PW641021* (QBG); Mae Sai Distr., Wat Phra Thard Doi thung, 1315 m, 22 May 2008, *V. Chamchumroon* et al. *1593* (BKF158522); Mae Suai Distr., Mae Suai, *K. Bunchuai & B. Nimanong 1473* (BKF36501); Doi Wa Wee, 1300 m alt., 1994, *Y. Prasorksantisatang s. n.* (BK62091); Ban San Sa At., 30 July 1967, *Prayad 952* (BK40805); Phan Distr., Wiang Bah Pao, Doi Luang National Park, 1300–1400 m alt., 28 October 1997, *J.F. Maxwell 97-1263* (BKF121813, L3896511); ibid., 1200 m alt, 24 May 1998, *J.F. Maxwell 98-557* (BKF121738, L3896507)]. Lampang [Hang Chat Distr., Khun Tarn, 10 February 1976, *Put 125* (BKF16254); Mae Tha Distr., Doi Khun Tan, 1000–1370 m, 27 December 1984, *H. Koyama & C. Phengklai T-39059* (BKF110946); ibid., *H. Koyama & C. Phengklai T-39059* (BKF110946); Mueang Pan Distr., Mueang Pan, Khun Chae National Park, 1427 m alt., 6 August 2020, *S. Ruchisansakun* et al. *PJCS63002* (QBG); *PJCS63004* (QBG); Chae Son, 1210 m alt, 9 November 2021, *S. Ruchisansakun* et al. *PW641109* (QBG)]. Lamphun [Mae Tha Distr., Tha Pla Duk, 17 June 2022, *S. Ruchisansakun MW650607* (QBG); Khun Tan, 1200 m alt., 2 November 1975, *T. Smitinand 12080* (BKF63463); ibid., 1050 m alt., 5 June 1993, *J.F. Maxwell 93-578* (BKF152332, L3808810)]. Mae Hong Son [Mae Hong Son, Khun Yuam Distr., Mae U Kho, 1240 m alt., 10 November 2021, *S. Ruchisansakun* et al. *PW641110* (QBG); ibid., 1360 m alt., 19 June 2022, *S. Ruchisansakun* et al. *PW641110* (QBG); 26 June 1998, *Chusie KY392* (QBG47309)]. Phayao [1510 m alt, 6 May 2015, *M. Norsaengsri 12354* (QBG82970); 1054 m alt., 31 June 2016, *N. Muangyen 1053* (QBG91043)].

*Vernacular name*. ตีนฮุ้งดอย สัตตฤาษี จ้วงนางแฮะ (Chiang Mai) ว่านตีนฮุ้ง (Lampang).

*Karyotype*. 2n = 2x = 10 = 6m + 4t, 6m + 4st, or 4m + 2sm + 4st [10].

*Note. Paris yunnanensis* is a cryptic species with very high variation in its morphology. The population in Thailand differs from *P. yunnanensis* in China [8] in many characters (as shown in Table 1), such as having petals above sepals (versus petals below sepals) and petals that are shorter than sepals (versus longer than or as long as sepals). The plant from the locality of *J.F. Maxwell 98-557*, which was identified as *P. cabangensis* [2], was also proven to be *P. yunnanensis*.

**Figure 1:**
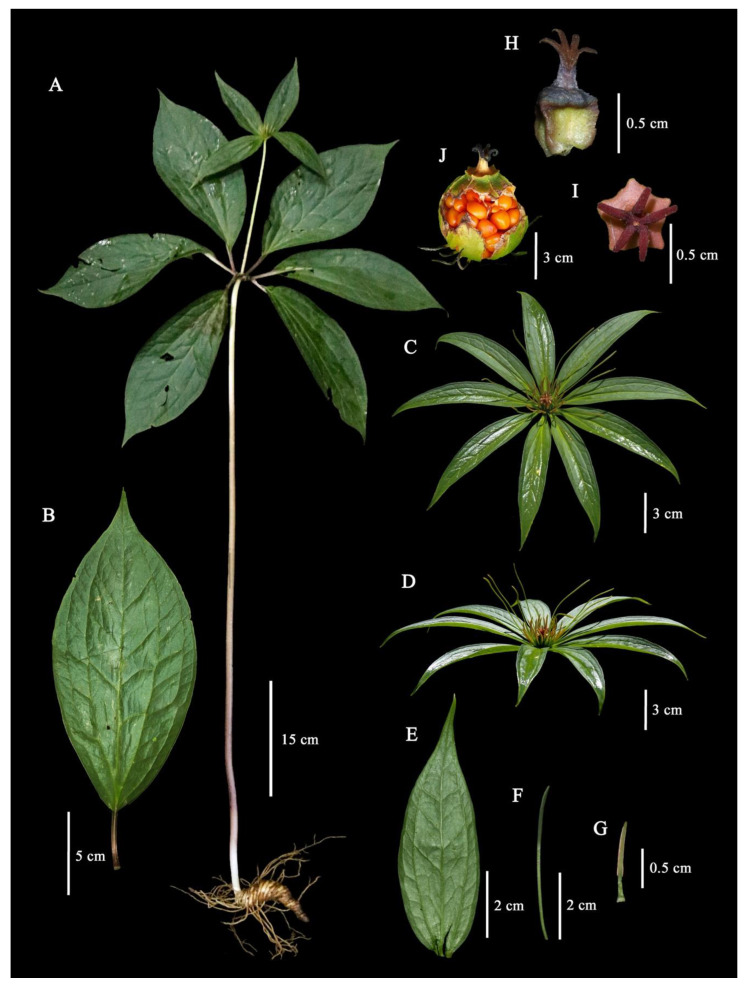
*Paris yunnanensis* Franch. (**A**) Habit, (**B**) Leaf, (**C**) Flower (front view), (**D**) Flower (lateral view), (**E**) Sepal, (**F**) Petal, (**G**) Stamen, (**H**) Pistil (front view), (**I**) Pistil (lateral view), (**J**) Fruit.

#### 2.1.2. *Paris siamensis* Ruchis. (Figure 2 and Figure 3)

*Paris siamensis* is similar to *P. liiana* but differs in having petals below sepals (*versus* petals above sepals), stamen number (2.2–)2.8–3 times petal number (*versus* stamen number 2 times petal number), and anther length 8–10 mm long (*versus* anther 15–40 mm long). Type: Thailand, Northern, Nan, Mae Charim Distr., Nong Daeng, 18°48′04.3″ N 101°12′47.0″ E, 1216 m alt, 21 April 2022, *P. Umpunjun* et al. *PW650421* (holotype QBG!, isotypes QBG!).

Perennial herb, 63.5–110(–158) cm tall. *Rhizome* thick, cylindrical, conical, to C-shaped, 9.5–15(–28.5) cm long, 2.5–4 cm in diam., glabrous, with 5–7 scars on the flowering state. *Aerial stems* (25–)–43–97 cm long, (0.85–)1–1.5 cm in diam., purple to green, or green at the upper part, glabrous to remotely short spiny. *Leaves* 6–7; *petiole* (4.5–)6–9 cm long, 3–4 mm in diam., purple above, glabrous; lamina 17–24 × 11–16 cm, ovate, elliptic, to obovate, base cuneate, to round, apex acute, width/length ratio 0.56–0.77: lateral veins two pairs. *Flower* solitary, terminal; *pedicel* 15.5–40(–61) cm long, 3–5 mm in diam., green to purple with green at the upper part, glabrous; *Sepals* 6–7, leaf-like, (6–)6.7–9.5 × 2.2–4.5 cm, ovate to narrowly ovate, width/length ratio 0.34–4.7, base obtuse to stipitate, apex acute, green, glabrous; *petals* 6–7, 2.6–5.4 cm × 1–3 mm, linear, protruding under the sepal, yellow to green: upper part 2.3–2.5 cm × 2–2.5 mm, petals/sepal length ratio (0.27–)0.65–0.75; *Stamen* (13–)17–21, stamens/petals number ratio (2.2–)2.8–3: filaments 4–7 mm long, ca. 1 mm in diam.: anthers 8–10 mm long: free portion of connective 1–1.5 × ca. 1 mm, yellow to brown, glabrous, anther/filament length ratio (1.14–)1.8–2.25. *Pistil* 1: style with an enlarged base: Stylar base purple to orange: Ovary 6–7 angled, single-locular with parietal placentation: stigma 6–7, ca 4 mm long, strongly curved, orange to red. Fruits dehiscent capsule, angular, 6–7 angled; immature fruit up to 9 mm long, up to 13 mm in diam., green to dark green, yellow at ovary base, glabrous. Seed white covered by red to orange: sarcotesta red to orange.

*Phenology*. Flowering April–August. Fruiting July–February.

*Distribution*. To date, endemic to Nan province, Thailand (may be found in Laos), 1050–1216 m alt.

*Vernacular name*. Tin Hung Doi Siam (ตีนฮุ้งดอยสยาม), Tin Hung Doi (ตีนฮุ้งดอย).

Additional Specimens Examined. THAILAND. NORTHERN. Nan.

[Bo Kluea Distr., Dong Phaya, 20 April 2022, *P*. *Umpunjun* et al. PW650420 (QBG);

Mae Charim Distr., Nong Daeng, 1216 m alt.; ibid., 750 m alt. 26 December 2016, *V. Nguanchoo* 1016 (QBG102719); Pua Distr., Phu Kha, Doi Phu Kha National Park, 1050 m alt, 19 October 2021, *S. Ruchisansakun* et al. PW641019 (QBG); ibid., 1100 m alt., *R. Pooma* 1098 (BKF102154); ibid., 1600 m alt., 10 April 1991, *R*. *Pooma* 467 (BKF97579); ibid., 1200 m alt., 2 July 1999, *P. Srisanga* et al. 837 (QBG14625); ibid., 1700 m alt., 11 December 2002, *P. Srisanga* 2657 (QBG23648); ibid., 1280 m alt., 19 June 2010, *K. Srithi* 491 (QBG65084)].

Karyotype. 2n = 2x = 10 = 6m + 4t [10].

Note. *Paris siamensis* is similar to *P*. *liiana*, *P*. *yunnanensis* (population in China), and *P*. *chinensis*. It differs from *P*. *liana*, as mentioned in the species diagnosis. Differences compared to other species are shown in Table 1.

**Figure 2:**
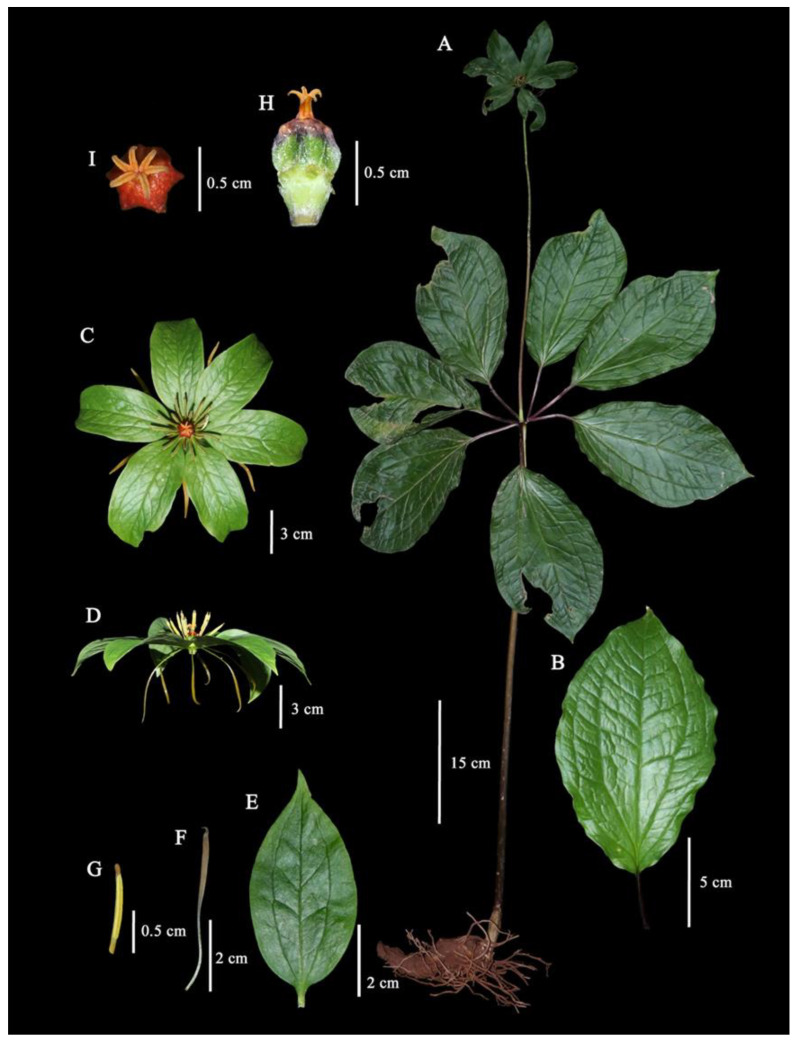
*Paris siamensis* Ruchis. (**A**) Habit, (**B**) Leaf, (**C**) Flower (front view), (**D**) Flower (lateral view), (**E**) Sepal, (**F**) Petal, (**G**) Stamen, (**H**) Pistil (front view), (**I**) Pistil (lateral view).

**Figure 3:**
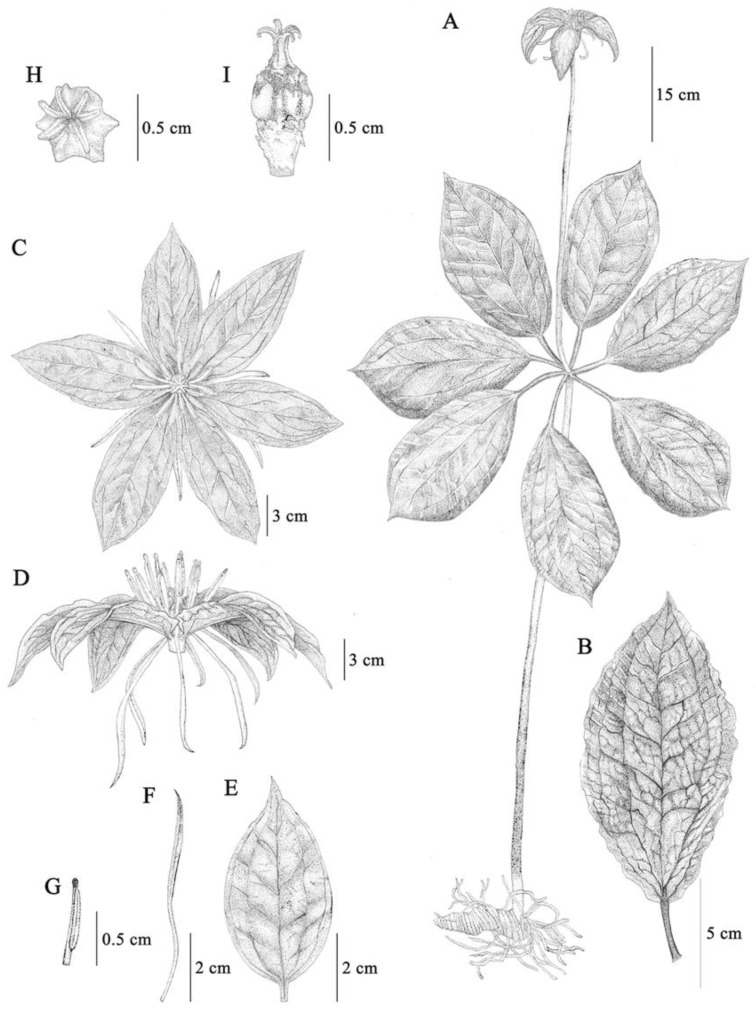
*Paris siamensis* Ruchis. (**A**) Plant, (**B**) Leaf, (**C**) Flower (front view), (**D**) Flower (lateral view), (**E**) Sepal, (**F**) Petal, (**G**) Stamen, (**H**) Pistil (front view), (**I**) Pistil (lateral view). (Drawn by Jeerapach Monthanom).

### 2.2. Phylogenetic Analysis

**Figure 4:**
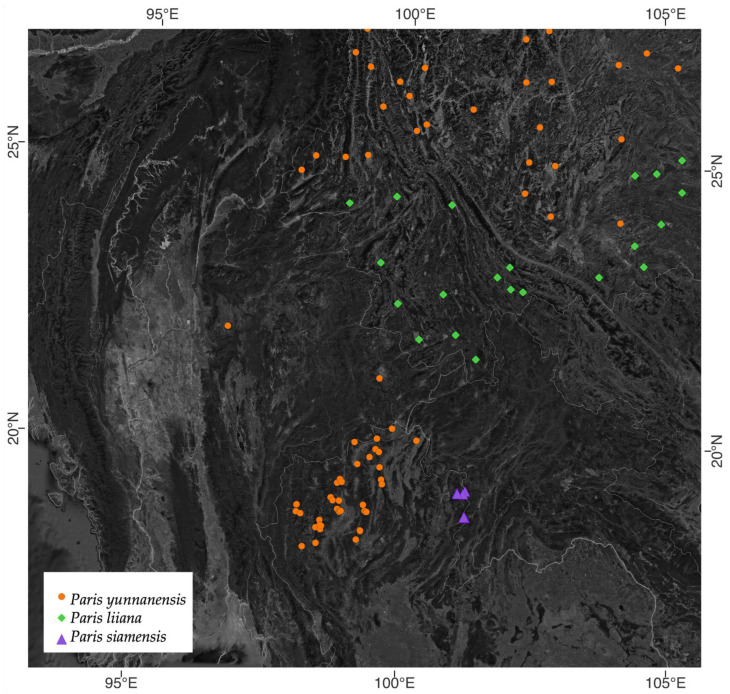
The distribution map of *P. yunnanensis*, *P. liiana*, and *P. siamensis*.

**Figure 5:**
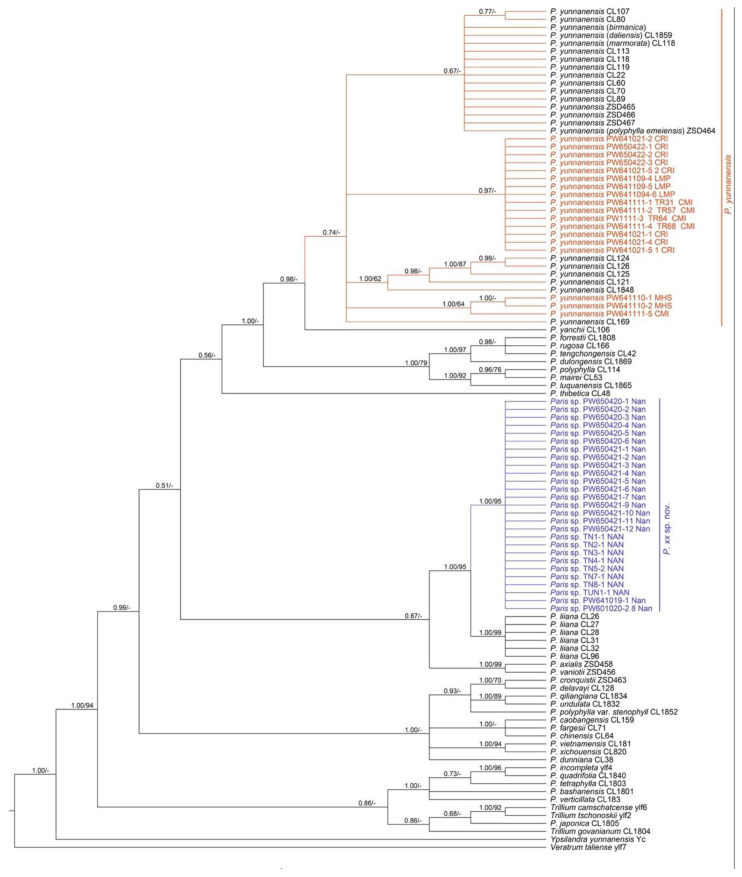
The majority rule consensus tree results from Bayesian inference for the ITS dataset. Numbers above branches indicate posterior probabilities (PP) and bootstrap percentage (BP). Dashes indicate support values of less than 50%. The two species of *Paris* from Thailand are distinguished by different colors.

Most samples of *Paris* in Thailand collected from Chiang Mai, Chiang Rai, Mae Hong Son, and Lampang provinces (Figure 4) were classified in the same clade as *P. yunnanensis* with a low support value of 0.74 (Figure 5). This clade was sister to *P. yanchii* with high support (PP = 0.98). All samples collected from Nan province, Thailand, were monophyletic with high support (PP = 1:ML-MP = 95). This clade was sister to *P. liiana* with high support (PP = 1:ML-MP = 95).

### 2.3. Differences between P. yunnanensis and P. siamensis in Thailand

#### 2.3.1. ANOSIM Analysis

The results of the similarity analysis (ANOSIM) showed that morphological characters between *P. yunnanensis* and *P. siamensis* were significantly different in terms of both flowering state (R-value = 0.3461, *p*-value = 0.0087) and non-flowering state (R-value = 0.0988, *p*-value = 0.0442).

#### 2.3.2. Principal Component Analysis (PCA) of Flowering State

The Kaiser–Meyer–Olkin (KMO) measure was used to verify sampling adequacy for the analysis. The obtained KMO value for the flowering state was 0.639, indicating that the sample size was adequate for factor analysis. Analysis of Bartlett’s test of sphericity (χ^2^(351)) was 2959.810 with a *p*-value < 0.001, suggesting that correlations between items were sufficiently large for PCA.

**Figure 6:**
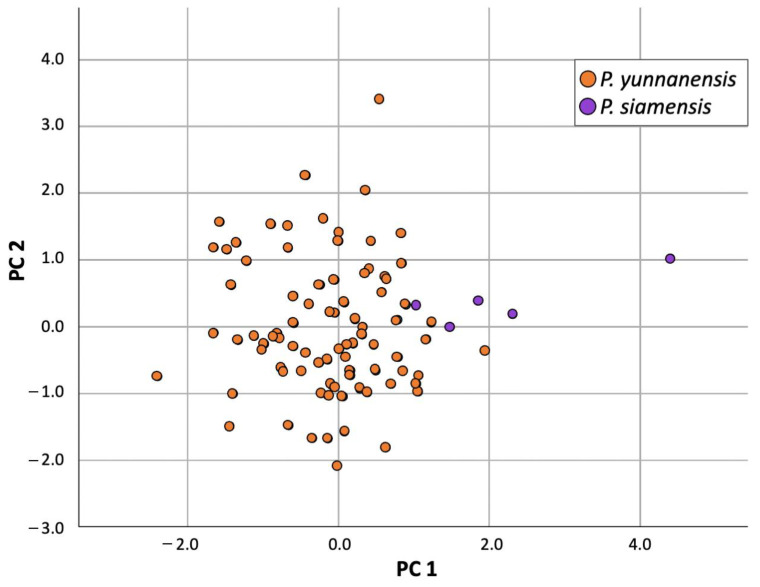
Principal Component Analysis results (PC1, PC2) of *P. yunnanensis* and *P. siamensis* in the flowering state.

The first two Principle Component axes explained 40.35% of the variation (Figure 6). PC1, which explained 12.40% of the variation, seemed to identify lamina width. PC2, which explained 27.95% of the variation, seemed to identify petiole length, lamina length, the number of sepals, sepal length, the number of petals, anther length, anther width, and ovary length. Axis PC2 was unable to separate the two species, while axis PC1 showed separation with some overlap. The values of *P. siamensis* along PC1 were greater than those of *P. yunnanensis*, indicating that *P. siamensis* generally differs from *P. chinensis* in terms of its greater lamina width.

#### 2.3.3. Principal Component Analysis (PCA) of Non-Flowering State

The Kaiser–Meyer–Olkin (KMO) value for the non-flowering state was 0.697, which is greater than 0.5, indicating that the sample size was adequate for factor analysis. Bartlett’s test of sphericity (χ^2^(21) = 344.429; *p*-value < 0.001) indicated that correlations between items were sufficiently large for PCA.

**Figure 7:**
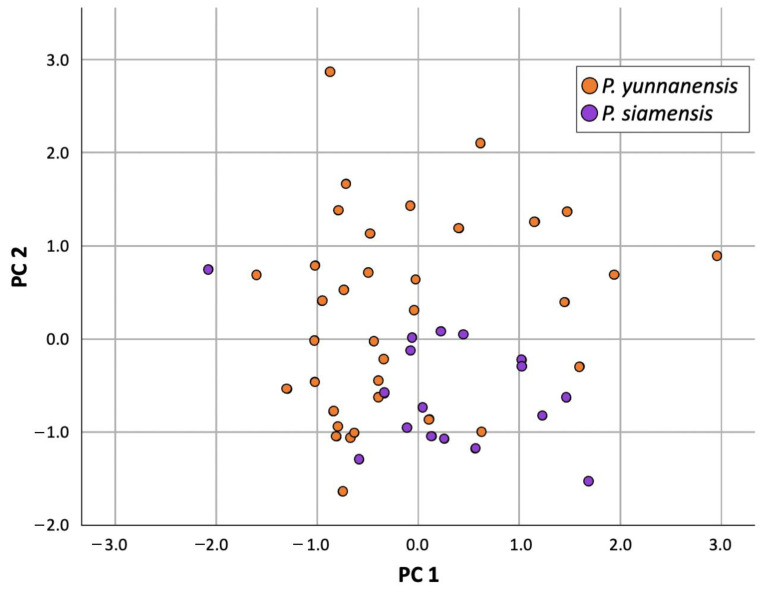
Principal Component Analysis results (PC1, PC2) of *P. yunnanensis* and *P. siamensis* in the non-flowering state.

The first two Principle Component axes explained 78.84% of the variation (Figure 7). PC1, which explained 61.81% of the variation, seemed to identify stem length, tuber length, petiole length, lamina length, lamina width, and the number of leaves. In addition, PC2, which explained 17.03% of the variation, appeared to identify the leaf width/length ratio. Axis PC1 was unable to separate the two species, while axis PC2 divided these two species with little overlap. The values of *P. siamensis* along PC2 were less than those of *P. yunnanensis*, indicating that *P. siamensis* generally differed from *P. yunnansis* in terms of their smaller leaf width/length ratio.

#### 2.3.4. Differences between *P. yunnanensis* and *P. siamensis*

**Figure 8:**
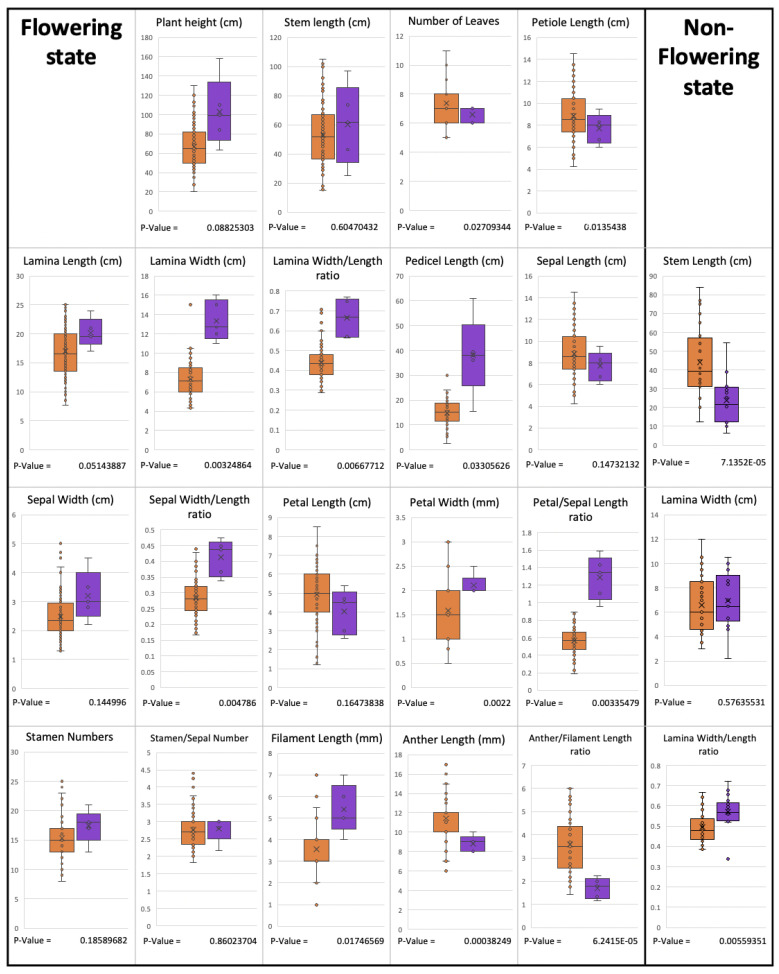
Comparison between some morphological characters of *P. yunnanensis* and *P. siamensis*. *p*-values were obtained from *t*-tests; brown represents *P. yunnanensis*, and violet represents *P. siamensis*.

The results from *t*-tests showed that these two species are different with respect to several characteristics in both the flowering and non-flowering stages (Figure 8). In the flowering stage, lamina width, lamina width/length ratio, sepal width/length ratio, etc., were significantly different between the two species. In the non-flowering stage, the lamina width/length ratio and stem length were also significantly different.

## 3. Discussion

### 3.1. The Morphology and Phylogenetic Position of Paris in Thailand

The genus *Paris* has not yet been well identified and still remains confusing due to high intra-specific variation [2]. Both species in Thailand look similar to other species, especially *P. chinensis*. Even though both species exhibit morphological differences, it is still difficult to identify them only by morphology. Fortunately, the molecular data of all known species in this genus has already been provided by Ji et al. [7,8]. In addition, our molecular phylogenetic tree is congruent with Ji et al. [7,8]. The new samples from Thailand are clearly separated into two clades. The first clade is *P. yunnanensis*. However, this clade is polytomous and received low support. The other clade consists of our samples from Nan province and is identified as a sister to *P. liiana* with high support.

### 3.2. The Distributions of the Two Paris Species in Thailand

The distributions of the plants showed that this genus is specific to high-altitude habitats. The two species in Thailand were separated by lowland areas along the Nan river. *Paris siamensis* was found only in Luang Prabang Rang, while *P. yunnanensis* was more widespread and found in the Phi Pan Nam Range, the Thong Chai Range, and the Daen Lao Range, which connects to the Shan plateau where the same species is found and is connected to the populations in China [2].

## 4. Materials and Methods

### 4.1. Plant Specimen Collection and Morphological Study

Morphological characters of *Paris* specimens were examined from living materials and herbarium specimens from BK, BKF, K, L, and QBG (abbreviations of the herbaria follow the Index Herbariorum). Intensive fieldwork was conducted in northern Thailand in the years 2020 to 2022. Most of the new voucher specimens were deposited in the QBG herbarium. The morphological characters of around 100 specimens were measured and recorded, mostly from living specimens. The terminology used when describing morphological characters follows Beentje and Williamson [11].

### 4.2. Molecular Phylogenetic Study

Forty-five *Paris* sequences from Thailand and 56 *Paris* sequences from Genbank database were used in the analysis, including every known species of *Paris* [2]. The species names were defined by Ji et al. [7,8]. In addition, *Trillium*, *Veratrum*, and *Ypsilandra* were chosen as outgroups (Table S1).

We used nuclear internal transcribed spacer (ITS) sequences to construct phylogenetic trees using Bayesian inference (BI) and Maximum likelihood (ML) methods. DNA alignment was performed by MUSCLE in MEGA. The best evolutionary model was constructed using jModelTest2 ver. 2.1.6. on XSEDE [12]. The GTR + I + G model was selected as the best-fit model based on Akaike information criterion (AIC) values.

The BI analyses were performed using MrBayes on XSEDE ver. 3.2.7a [13] via CIPRES [14]. Two independent parallel runs of 4 simultaneous Markov Chain Monte Carlo (MCMC) algorithms (containing 3 heated chains and 1 cold chain with a 0.2 temperature value) were performed for 10,000,000 generations, and trees were sampled every 100 generations. The initial 25% of sampled trees were discarded. The 50% majority rule consensus tree and posterior probability (PP) were calculated from the remaining trees. Convergence of independent runs was assessed by checking the standard deviation of split frequencies with values <0.01, Estimated sample size (ESS) with values >200, and Potential scale reduction factor (PSRF) with values approaching 1.0. The ML analyses were conducted using RAxML-HPC BlackBox v8.1.24 with 1000 replicates of rapid bootstrapping. All phylogenetic trees were visualized with FigTree v.1.4.4 [15].

### 4.3. Statistical Analysis of the Differences in Morphological Characters of the Species in Thailand

Descriptive statistics for all factors were analyzed with SPSS 18 and used to calculate means and standard deviations. *t*-tests were used to examine significant differences between the two *Paris* species for some morphological characters of interest. The differences in morphological characters between *P. yunnanensis* and *P. siamensis* were investigated using multivariate analyses of similarity (ANOSIM) and principal component analysis (PCA). ANOSIM was performed with R programming (v. 1.4.1717). This analysis is used to test for significant differences between two or more groups of sampling units [16].

PCA was analyzed by SPSS 18 to extract patterns for differentiating between plant species. Variation in quantitative characters was visualized using PCA. The variance-covariance matrix was calculated based on the quantitative characters, and the first two principal components were plotted.

## Figures and Tables

**Table 1:** Comparison of the morphology of *P. siamensis* and related species.

	*P*. *siamensis* (Ruchis. et al.)	*P*. *liiana* [8]	*P*. *chinensis* [2]	*P*. *yunnanensis* [8]	*P*. *yunnanensis* (Thailand)	*P. caobangensis* [2]	*P. vietnamensis* [2]
Lamina width (cm)	11–16	8–15	2–8	3–7	4.3–10.5(–15)	ca. 4.5	11–16
Lamina width/length ratio	0.56–0.77	ca 0.4–0.5	ca 0.25–0.4	ca 0.38–0.47	0.29–0.71	0.47	0.6–0.62
Sepal width (cm)	2.2–4.5	2.5–5	0.8–3	1.5–3	1.3–5	1.5–2.5	1–4
Petal	Below calyx	Above calyx	Below calyx	Below calyx	Above calyx	Below calyx	Above calyx
Petal length/Sepal length ratio	(0.27)–0.65–0.75	Shorter or slightly longer than sepals	Much shorter (occasionally slightly longer) than sepals	Longer than or as long as sepals	(0.19)–0.33–0.89	Longer than sepals	Longer than or as long as sepals
Stamens/Petals number ratio	(2.2–)2.8–3	ca 2	ca 2	ca 2	(1.83–)2–3.2(–4.4)	2	2 or 3
Anther length (mm)	8–10	15–40	5–10	5–15	7–13(–17)	6–9	8–13
Anther length/filament length ratio	(1.1–)1.8–2.25	5–6.7	1.67–1.43	1.5–1.67	(1.43–)2–6	0.38–0.47	1.3–2

## Data Availability

All relevant data can be found within the manuscript and Supplementary Materials.

## Supplementary Materials

The following supporting information can be downloaded at: https://www.mdpi.com/article/10.3390/plants12030430/s1, Table S1: Voucher and Genbank accession numbers information used in the molecular analyses.

## References

[1] Linnaeus C. (1753). Species plantarum 2. Impensis Laurentii Salvii.

[2] Ji Y.H. (2021). Taxonomic revision. A Monograph of Paris (Melanthiaceae).

[3] Trias Blasi A., Parnell J., Watson M. (2017). Nomenclatural notes on species of Asian Vitaceae. Taxon.

[4] Franchet A. (1888). Monographie du genre *Paris*. Mémoires Publiés Par La Société Philomath..

[5] Ji Y.H., Li H., Zhou Z.K. (2006). *Paris caobangensis* Y. H. Ji, H. Li & Z.K. Zhou (*Trilliaceae*), a new species from northern Vietnam. J. Syst. Evol..

[6] Ji Y.H., Fritsch P.W., Li H., Xiao T., Zhou Z. (2006). Phylogeny and classification of *Paris* (*Melanthiaceae*) inferred from DNA sequence data. Ann. Bot..

[7] Ji Y.H., Yang L.F., Chase M.W., Liu C.K., Yang Z.Y., Yang J., Yang J.-B., Yi T.-S. (2019). *Plastome phylogenomics*, biogeography, and clade diversification of *Paris* (*Melanthiaceae*). BMC Plant Biol..

[8] Ji Y., Liu C., Yang J., Jin L., Yang Z., Yang J.-B. (2020). Ultra-barcoding discovers a cryptic species in *Paris yunnanensis* (*Melanthiaceae*), a medicinally important plant. Front. Plant Sci..

[9] Zhengyi W., Raven P.H. (2000). Flora of China 24: 1-431.

[10] Chowa J., Puangpairoteb T., Anamthawat-Jónssonc K., Umpunjuna P. (2020). Karyotypic and molecular cytogenetic characterization of diploid and polyploid accessions of medicinal herbs in the genus *Paris* from northern Thailand. Scienceasia.

[11] Beentje H., Williamson J. (2010). The Kew Plant Glossary: An Illustrated Dictionary of Plant Terms.

[12] Darriba D., Taboada G., Doallo R., Posada D. (2012). jModelTest 2: More models, new heuristics and parallel computing. Nat. Methods.

[13] Ronquist F., Teslenko M., van der Mark P., Ayres D., Darling A., Höhna S., Larget B.R., Liu L., Suchard M.A., Huelsenbeck J.P. (2012). MrBayes 3.2: Efficient Bayesian phylogenetic inference and model choice across a large model space. Syst. Biol..

[14] Miller M., Pfeiffer W., Schwartz T. (2010). Creating the CIPRES Science Gateway for Inference of Large Phylogenetic Trees.

[15] Rambaut A. (2018). Retrieved from FigTree, Version 1.4.4. https://github.com/rambaut/figtree/releases.

[16] Clarke K.R. (1993). Non-parametric multivariate analysis of changes in community structure. Aust. J. Ecol..

