# Supplementary material for: Revision on the Genus Paris in Thailand, with a New Species Paris siamensis
Source: Plants (Basel). 2023 Jan 17;12(3):430. doi: 10.3390/plants12030430 (PMC9919091; doi:10.3390/plants12030430)
Supplement: Supplementary file 1 [file plants-12-00430-s001.zip › plants-2082175-supplementary.pdf]

Table S1 Voucher and GenBank accession numbers information used in the molecular analyses.

Taxon	Locality	Voucher	GenBank accession number
Paris axialis H.Li	China, Yunnan, Zhaotong	Y. Ji s. n. (ZSD458)	MN174902
P. bashanensis F.T.Wang & Tang	China, Sichuan, Pengzhou	H. Liang and X. Zhang 1332 (CL1801)	MN174874
P. yunnanensis Franch. (*P. birmanica* (Takht.) H.Li & Noltie)	Myanmar, Introduced from northern Myanmar	Y. Ji 2016433 (CL89)	MN174895
P. caobangensis Y. H. Ji, H. Li et Z. K. Zhou	China, Yunnan, Guangnan	Y. Ji 2016618 (CL159)	MN174871
P. cronquistii (Takht.) H. Li	China, Sichuan, Wanyuan	Y. Ji et al. s. n. (ZSD463)	MN174903
P. daliensis H. Li & V. G. Soukup	China, Yunnan, Lanping	Y. Ji et al. 1859 (CL1859)	MN174885
***P. delavayi* Franch.**	Chinan, Yunnan, Daguan	Y. Ji 2016519 (CL128)	MN174870
P. dulongensis H.Li & Kurita	China, Yunnan, Gongshan	H. Li 57 (CL1869)	MN174887
P. dunniana Lévl.	China, Hainan, Wuzhishan	HYL–34 (CL38)	MN174888
P. fargesii Franch.	China, Hanan, Sangzhi	Y. Ji 2016414 (CL71)	MN174893

P. forrestii (Takht.) H.Li	China, Yunnan, Gongshan	G. Zhou s.n. (CL1808)	MN174877
P. incompleta M. Bieb.	Georgia	Z. Zhou and H. Sun s.n. (ylf4)	MN174898
P. japonica Franch. & Sav.) Franch.	Edinburgh, Cult. in Royal Botanical Garden	1974145813B (CL1805)	MN174876
P. liiana Y. H. Ji	China, Yunnan, Qiubei	HYL06 (cl26)	MN647563
P. liiana Y. H. Ji	China, Yunnan, Xichou	HYL12 (Cl31)	MN647566
P. liiana Y. H. Ji	China, Yunnan, Mojiang	HYL13 (Cl32)	MN647567
P. liiana Y. H. Ji	China, Yunnan, Yuanyang	JYH2016457 (Cl96)	MN647572
P. liiana Y. H. Ji	China, Yunnan, Xinping	HYL07 (Cl27)	MN647564
P. liiana Y. H. Ji	China, Yunnan, Jinghong	HYL08 (Cl28)	MN647565
P. luquanensis H. Li	China, Yunnan, Luquan	Y. Ji et al. 1865 (CL1865)	MN174886
P. mairei Lévl.	China, Yunnan, Lijiang	Y. Ji 20160392 (CL53)	MN174891
P. marmorata Stearn	China, Yunnan, Shangri-La	Y. Ji 2016503 (CL118)	MN174869
P. chinensis Franch.	China, Hubei, Enshi	Y. Ji 2016407 (CL64)	MN174892
P. *polyphylla* var. *stenophylla* Franch.	China, Sichuan, Luding	Y. Ji 2018051 (CL1852)	MN174884
P. quadrifolia L.	Netherlands	P. Bruggeman s. n. (CL1840)	MN174882
P. rugosa H.Li & Kurita	China, Yunnan, Changning	Y. Ji 2016525 (CL166)	MN174872

P. siamensis Ruchis.	Thailand, Nan	P. Umpunjun et al. PW601020-2	OP296616
P. siamensis Ruchis.	Thailand, Nan, Pua, Phu Kha	S. Ruchisansakun et al. PW641019-1	OP296617
P. siamensis Ruchis.	Thailand, Nan, Bo Kluea	P. Umpunjun et al. PW650420-1	OP296618
P. siamensis Ruchis.	Thailand, Nan, Bo Kluea	P. Umpunjun et al. PW650420-2	OP296619
P. siamensis Ruchis.	Thailand, Nan, Bo Kluea	P. Umpunjun et al. PW650420-3	OP296620
P. siamensis Ruchis.	Thailand, Nan, Bo Kluea	P. Umpunjun et al. PW650420-4	OP296621
P. siamensis Ruchis.	Thailand, Nan, Bo Kluea	P. Umpunjun et al. PW650420-5	OP296622
P. siamensis Ruchis.	Thailand, Nan, Bo Kluea	P. Umpunjun et al. PW650420-6	OP296623
P. siamensis Ruchis.	Thailand, Nan, Mae Charim	P. Umpunjun et al. PW650421-1	OP296624
P. siamensis Ruchis.	Thailand, Nan, Mae Charim	P. Umpunjun et al. PW650421-2	OP296625
P. siamensis Ruchis.	Thailand, Nan, Mae Charim	P. Umpunjun et al. PW650421-3	OP296626
P. siamensis Ruchis.	Thailand, Nan, Mae Charim	P. Umpunjun et al. PW650421-4	OP296627
P. siamensis Ruchis.	Thailand, Nan, Mae Charim	P. Umpunjun et al. PW650421-5	OP296628
P. siamensis Ruchis.	Thailand, Nan, Mae Charim	P. Umpunjun et al. PW650421-6	OP296629
P. siamensis Ruchis.	Thailand, Nan, Mae Charim	P. Umpunjun et al. PW650421-7	OP296630
P. siamensis Ruchis.	Thailand, Nan, Mae Charim	P. Umpunjun et al. PW650421-9	OP296631

P. siamensis Ruchis.	Thailand, Nan, Mae Charim	P. Umpunjun et al. PW650421-10	OP296632
P. siamensis Ruchis.	Thailand, Nan, Mae Charim	P. Umpunjun et al. PW650421-11	OP296633
P. siamensis Ruchis.	Thailand, Nan, Mae Charim	P. Umpunjun et al. PW650421-12	OP296634
P. siamensis Ruchis.	Thailand, Nan, Mae Charim	P. Umpumchan et al. TN1-1	OP296635
P. siamensis Ruchis.	Thailand, Nan, Mae Charim	P. Umpumchan et al. TN2-1	OP296636
P. siamensis Ruchis.	Thailand, Nan, Mae Charim	P. Umpumchan et al. TN3-1	OP296637
P. siamensis Ruchis.	Thailand, Nan, Mae Charim	P. Umpumchan et al. TN4-1	OP296638
P. siamensis Ruchis.	Thailand, Nan, Mae Charim	P. Umpumchan et al. TN5-2	OP296639
P. siamensis Ruchis.	Thailand, Nan, Mae Charim	P. Umpumchan et al. TN7-1	OP296640
P. siamensis Ruchis.	Thailand, Nan, Mae Charim	P. Umpumchan et al. TN8-1	OP296641
P. siamensis Ruchis.	Thailand, Nan, Mae Charim	P. Umpumchan et al. TUN101	OP296642
P. tengchongensis Y.H.Ji et. al.	China, Yunnan, Tengchong	Y. Ji 361 (CL42)	MN174889
P. tetraphylla A.Gray	Japan	K. Inoue and T. Kubo 1609 (CL1803)	MN174875
P. thibetica Franch.	China, Yunnan, Tengchong	Y. Ji 20160387 (CL48)	MN174890
P. undulata H.Li & V.G.Soukup	China, Sichuan, Emeishan	Y. Ji et al. 1832 (CL1832)	MN174879
***P. vaniotii* H.Lév**	China, Hunan, Xinning	H. Li 052 (ZSD456)	MN174901

P. verticillata M. Bieb.	China, Hebei, Chicheng	B. Liu 3490 (CL1837)	MN174881
P. vietnamensis (Takht.) H.Li	China, Yunnan, Jinping, Yunnan, China	Y. Ji et al. 1810 (CL1810)	MN174878
P. xichouensis H. Li.	China, Yunnan, Malipo	Y. Ji et al. 1820 (CL820)	MN174894
P. yanchii H. Li et al.	China, Yunnan, Jianchuan	Y. Ji 2016482 (CL106)	MN174868
P. yunnanensis Franch.	China, Yunnan, Wuding	Y. Ji 626 (CL169)	MN174873
P. yunnanensis Franch.	China, Yunnan, Chuxiong	HYL02 (cl22)	MN647562
P. yunnanensis Franch.	China, Yunnan, Dongchuan	JYH2016503 (Cl118)	MN647576
P. yunnanensis Franch.	China, Sichuan, Huidong	JYH2016424 (cl80)	MN647570
P. yunnanensis Franch.	China, Sichuan, Huili	JYH2016489 (cl107)	MN647573
P. yunnanensis 2020 Huize v	China, Yunnan, Huize	JYH2016403 (cl60)	MN647568
P. yunnanensis Franch.	China, Yunnan, Lanping	JYH2016516 (cl125)	MN647580
P. yunnanensis Franch.	China, Yunnan, Luquan	JYH2016504 (cl119)	MN647577
P. yunnanensis Franch.	China, Sichuan, Muli	JYH2016515 (cl124)	MN647579
P. yunnanensis Franch.	Myanmar, Waingmaw	JYH2016433 (cl89)	MN647571
P. yunnanensis Franch.	China, Yunnan, Shangri-la	JYH2016517 (cl126)	MN647581
P. yunnanensis Franch.	China, Yunnan, Ninglang	JYH2016498 (cl113)	MN647574

P. yunnanensis Franch.	China, Sichuan, Mianning	JYH2017039 (zsd465)	MN647582
P. yunnanensis Franch.	China, Sichuan, Yanyuan	JYH2017046 (zsd466)	MN647583
P. yunnanensis Franch.	China, Sichuan, Shimian	JYH2017041 (zsd467)	MN647584
P. yunnanensis Franch.	China, Yunnan, Weishan	JYH2016413 (cl70)	MN647569
P. yunnanensis Franch.	China, Yunnan, Yongping	JYH2016507 (cl121)	MN647578
P. yunnanensis Franch.	Thailand, Chiang Mai, Chai Prakan	S. Ruchisansakun et al. PW641111-1	OP296643
P. yunnanensis Franch.	Thailand, Chiang Mai, Chai Prakan	S. Ruchisansakun et al. PW641111-2	OP296644
P. yunnanensis Franch.	Thailand, Chiang Mai, Chai Prakan	S. Ruchisansakun et al. PW641111-3	OP296645
P. yunnanensis Franch.	Thailand, Chiang Mai, Chai Prakan	S. Ruchisansakun et al. PW641111-4	OP296646
P. yunnanensis Franch.	Thailand, Chiang Mai, Chai Prakan	S. Ruchisansakun et al. PW641111-5	OP296647
P. yunnanensis Franch.	Thailand, Chiang Rai, Mae Fa Luang	P. Umpunjun et al. PW650422-1	OP296648
P. yunnanensis Franch.	Thailand, Chiang Rai, Mae Fa Luang	P. Umpunjun et al. PW650422-2	OP296649
P. yunnanensis Franch.	Thailand, Chiang Rai, Mae Fa Luang	P. Umpunjun et al. PW650422-3	OP296650
P. yunnanensis Franch.	Thailand, Chiang Rai, Mueang Chiang Rai	S. Ruchisansakun et al. PW641021-1	OP296651
P. yunnanensis Franch.	Thailand, Chiang Rai, Mueang Chiang Rai	S. Ruchisansakun et al. PW641021-2	OP296652
P. yunnanensis Franch.	Thailand, Chiang Rai, Mueang Chiang Rai	S. Ruchisansakun et al. PW641021-4	OP296653

P. yunnanensis Franch.	Thailand, Chiang Rai, Mueang Chiang Rai	S. Ruchisansakun et al. PW641021-5/1	OP296654
P. yunnanensis Franch.	Thailand, Chiang Rai, Mueang Chiang Rai	S. Ruchisansakun et al. PW641021-5/2	OP296655
P. yunnanensis Franch.	Thailand, Lampang, Mueang Pan	S. Ruchisansakun et al. PW641109-4	OP296656
P. yunnanensis Franch.	Thailand, Lampang, Mueang Pan	S. Ruchisansakun et al. PW641109-5	OP296657
P. yunnanensis Franch.	Thailand, Lampang, Mueang Pan	S. Ruchisansakun et al. PW641109-6	OP296658
P. yunnanensis Franch.	Thailand, Mae Hong Son, Khun Yuam	S. Ruchisansakun et al. PW641110-1	OP296659
P. yunnanensis Franch.	Thailand, Mae Hong Son, Khun Yuam	S. Ruchisansakun et al. PW641110-2	OP296660
P. *polyphylla* var. *emeiensis* H. X. Yin et al.	China, Sichuan, Ya'an	Y. Ji et al. s. n. (ZSD464)	MN174904
P. qiliangiana H. Li et al.	China, Sichuan, Xuanhan	Y. Ji et al. 1834 (CL1834)	MN174880
P. polyphylla Smith	China, Yunnan, Jianchuan	L. Yang 1848 (CL1848)	MN174883
T. camschatcense Ker Gawl.	China, Jilin, Panshi	H. An 090 (ylf6)	MN174899
T. govanianum Wall. ex D. Don	China, Tibet, Dingri	H. Li and S. Chen 391 (CL1804)	MN174867
T. tschonoskii Maxim.	China, Yunnan, Shangri-La	YLF–95 (ylf2)	MN174897
V. taliense O. Loes.	China, Yunnan, Gongshan	MN174900 (ylf7)	MN174900
Y. yunnanensis W. W. Sm. & Jeffrey	China, Yunnan, Fugong	GLGS Expedition 33923 (Yc)	MN174896
